# STAT3 Activation in Combination with NF-KappaB Inhibition Induces Tolerogenic Dendritic Cells with High Therapeutic Potential to Attenuate Collagen-Induced Arthritis

**DOI:** 10.1155/2019/1982570

**Published:** 2019-07-01

**Authors:** Carolina Prado, Valentina Ugalde, Hugo González, Alicia Figueroa, Ernesto López, Alvaro Lladser, Rodrigo Pacheco

**Affiliations:** ^1^Laboratorio de Neuroinmunología, Fundación Ciencia & Vida, Santiago 7780272, Chile; ^2^Laboratorio de Inmunoterapia Génica, Fundación Ciencia & Vida, Santiago 7780272, Chile; ^3^Departamento de Ciencias Biológicas, Facultad de Ciencias de la Vida, Universidad Andres Bello, Santiago 8370146, Chile

## Abstract

Dendritic cells (DCs) have the ability to induce tolerance or inflammation in response to self-antigens, which makes them fundamental players in autoimmunity. In this regard, immunogenic DCs produce IL-12 and IL-23 favouring the acquisition of Th1 and Th17 inflammatory phenotypes, respectively, by autoreactive CD4^+^ T-cells, thus promoting autoimmunity. Conversely, tolerogenic DCs produce IL-10 and TGF-*β*, inducing the generation of CD4^+^ T-cells with suppressive activity (Treg), which promote tolerance to self-constituents. Previous studies have shown that STAT3 signalling in DCs attenuates the production of proinflammatory cytokines, whilst NF-*κ*B activation promotes it. In this study, we aimed to generate DCs displaying strong and constitutive tolerogenic profile to be used as immunotherapy in autoimmunity. To this end, we transduced bone marrow-derived DCs with lentiviral particles codifying for a constitutively active version of STAT3 (constitutively active STAT3 (STAT3ca)) or with a constitutive repressor of NF-*κ*B (I*κ*B*α* superrepressor (I*κ*B*α*SR)), and their therapeutic potential was evaluated in a mouse model of arthritis induced by collagen (CIA). Our results show that STAT3ca transduction favoured the production of the anti-inflammatory mediator IL-10, whereas I*κ*B*α*SR transduction attenuated the expression of the proinflammatory cytokine IL-23 in DCs. Moreover, both STAT3ca-transduced and I*κ*B*α*SR-transduced DCs separately exerted a mild but significant therapeutic effect reducing the severity of CIA development. Furthermore, when DCs were transduced with both STAT3ca and I*κ*B*α*SR together, they reduced CIA manifestation significantly stronger than when transduced with only STAT3ca or I*κ*B*α*SR separately. These results show STAT3 and NF-*κ*B as two important and complementary regulators of the tolerogenic behaviour of DCs, which should be considered as molecular targets in the design of DC-based suppressive immunotherapies for the treatment of autoimmune disorders.

## 1. Introduction

CD4^+^ T-cells constitute central players of adaptive immunity, as they might regulate the differentiation and function of several subsets of leukocytes, including not only cells of the innate immunity but also CD8^+^ T-cells and B-cells [1–4]. Thus, CD4^+^ T-cells lead the elimination of pathogens and tumours. It is noteworthy that autoreactive T-cells that leak from thymic negative selection reach the periphery and become part of the repertoire of naive T-cells. It has been proposed that this low degree of self-reactivity is physiologic and necessary for efficient tumour immunosurveillance [5, 6].

Importantly, dendritic cells (DCs) constitute key players triggering CD4^+^ T-cell-mediated immunity, as they are antigen-presenting cells (APCs) specialized in inducing the activation and differentiation of naive CD4^+^ T-cells [7]. In the absence of danger signals, such as substances that stimulate pattern recognition receptors (PRRs) or inflammatory cytokines [8, 9], DCs capture autoantigens in healthy tissues, process them, acquire tolerogenic features, and present the processed autoantigens to autoreactive naive CD4^+^ T-cells inducing their elimination or favouring their differentiation toward regulatory T-cells (Treg), thus promoting active tolerance to self-constituents [10–12]. According to the key role of DCs inducing active tolerance to autoantigens, the permanent depletion of DCs results in spontaneous autoimmunity [13].

Conversely, the stimulation of PRRs or the recognition of molecular cues associated to tissue damage by DCs promotes the acquisition of immunogenic features, involving molecular and functional changes which make DCs specialized for inducing differentiation of naive CD4^+^ T-cells toward the most appropriate effector phenotype, including T-helper 1 (Th1), Th2, or Th17 [14, 15]. Of note, Th1 and Th17 cells have been extensively described to be the phenotypes of autoreactive T-cells involved in autoimmunity [16, 17]. Thus, during the onset of autoimmune disorders, DCs seem to be fundamental for the activation of self-reactive T-cells that have leaked from thymic negative selection [18].

The dual and antagonistic ability of DCs to induce tolerance or inflammation to autoantigens makes these cells key players in triggering autoimmune disorders. Depending on the inflammatory context and the expression of cell-intrinsic regulators, DC-mediated presentation of autoantigens might promote or inhibit autoimmune responses. In this regard, a major inducer of the inflammatory behaviour of DCs is the nuclear factor kappa-light-chain-enhancer of activated B-cells (NF-*κ*B), which might be activated by a number of danger signals, including the stimulation of the inflammasome, PRR agonists, and proinflammatory cytokines [9]. Of note, NF-*κ*B activation in DCs promotes the secretion of IL-12 and IL-23, which play a fundamental role in the induction of Th1- and Th17-mediated immunity, respectively [19, 20]. On the other hand, it has been reported that the B7 ligand CTLA4, which is expressed on the Treg surface, triggers the phosphorylation and consequent activation of the signal transducer and activator of transcription 3 (STAT3) in DCs inducing a tolerogenic behaviour [21]. In this regard, activated STAT3 might attenuate the recruitment of NF-*κ*B to the promoter of p40 [22], a subunit shared by IL-12 and IL-23. In addition, activated STAT3 inhibits the action of the positive transcription elongation factor *b* on the promoter of IL-12p35 [23]. Thus, STAT3 and NF-*κ*B seem to be two key regulators of DC behaviour, which might define an immunogenic or tolerogenic profile in response to self-antigens.

Rheumatoid arthritis (RA) is a chronic autoimmune disorder occurring in about 5% of the general population, leading to severe joint damage and disability [24]. Previous evidence has shown that Th1 and Th17 cells are the phenotypes of autoreactive CD4^+^ T-cells involved in RA and its mouse model, the type II collagen-induced arthritis (CIA) [25, 26]. Of note, due to that functional phenotype of DCs represents a key factor in the decision to promote tolerance or inflammation in response to specific antigens, the usage of autologous tolerogenic DCs loaded with autoantigens as therapy for the treatment of RA has been recently tested in phase I clinical trials and proven to be safe [27, 28].

Since the administration of autologous tolerogenic DCs has been shown to be safe in humans in a number of clinical trials for RA and other autoimmune disorders [29], this represents a promising strategy to develop antigen-specific immunosuppressive therapies geared to avoid pathogenic inflammation without affecting the beneficial immune response. Interestingly, a group of studies performed in animal models has shown lentiviral transduction as a potent tool to insert immunosuppressive genes or to knock down proinflammatory features in DCs. In this way, the adoptive transfer of tolerogenic DCs generated by lentiviral transduction has been shown to exert a potent antigen-specific immunosuppressive therapy not only ameliorating autoimmune disorders but also attenuating allograft rejection and systemic inflammation associated with septic shock [30–33]. According to the potent and stable phenotype acquired by DCs upon lentiviral transduction and considering the important role of STAT3 and NF-*κ*B as regulators of DC behaviour, in this study, we aimed to test the therapeutic potential of the permanent NF-*κ*B inhibition and constitutive STAT3 activation given by lentiviral transduction to DCs, each alone or both together, in a mouse model of CIA.

## 2. Materials and Methods

### 2.1. Lentiviral Particle Generation

Constitutively active STAT3 (STAT3ca, Cys substitutions at Ala662 and Asn664) and cDNA were cloned in pLNCX vector and kindly provided by Dr. Robert Arceci [34]. For the construction of the lentiviral vector codifying STAT3ca, mouse STAT3ca sequence was excised from pLNCX vector and subcloned into a pLVX-IRES-ZsGreen1 vector (Clontech). To induce the permanent inhibition of NF-*κ*B, a lentiviral vector Ubc.PGK.Cre codifying for a nonphosphorylatable version of I*κ*B*α* containing the S32A and S36A mutations (I*κ*B*α* superrepressor (I*κ*B*α*SR)) was used (Ubc.IkBSR-Flag_PGK.Cre) [35]. To induce the expression of both STAT3ca and I*κ*B*α*SR, we generated a master viral vector using the pLVX plasmid encoding the CMV promoter at 5′, followed by murine I*κ*B*α*SR and STAT3ca separated by a P2A sequence, and codifying an IRES-ZsGreen1 at 3′ (pLVX-VMmu). Recombinant lentiviruses were produced by transfecting HEK293T with the lentiviral expression plasmids (pLVX-IRES-ZsGreen1, Ubc.PGK.Cre, pLVX-STAT3ca, Ubc.IkBSR-Flag_PGK.Cre, or pLVX-VMmu) and the packaging plasmids pCMV-dR8.91 and pCMV-VSV-G using TurboFect (Thermo fisher) according to the manufacturer's instructions. Cells were incubated for 48 h, and then, the supernatant was collected and fresh medium was added. 72 h later, the supernatant was collected again. Collected supernatants were gently mixed, centrifuged at 28,000 rpm at 4°C for 75 min. Pellets containing viral particles were stored at -80°C for use in subsequent experiments. Viral titer was performed as previously described [36].

### 2.2. DC Generation and Transduction

DCs were generated from 7- to 8-week-old male DBA/1J mice obtained from The Jackson Laboratory (Bar Harbor, ME). Bone marrow precursors were differentiated to DCs as described [37] and transduced with lentiviral particles on days 4 and 5 of differentiation at MOI 10 in the presence of 2 *μ*g/ml polybrene as indicated before [38]. Briefly, bone marrow precursors were grown in RPMI 1640 medium supplemented with 10% heat-inactivated FBS (both from Gibco, Life Technologies, CA) and 10 ng/ml recombinant mouse GM-CSF (PeproTech, NJ). On day 4, differentiation of DCs was routinely assessed obtaining >80% CD11c^+^ cells. At this time point, DCs were harvested for lentiviral infection. 10^6^ DCs/well were plated on 24-well plates at 37°C and 10^7^ pfu viruses were added at day 4 and at day 5 in a total volume of 1 ml of serum-free medium in the presence of 2 *μ*g/ml polybrene. Transduced DCs were recovered at day 6 and then analyzed in vitro or injected into animals. Routine immunophenotyping was performed by flow cytometry analysis using the following fluorochrome-conjugated monoclonal antibodies: anti-CD11c-APC (N418), anti-CD86-PE (GL1), anti-PDL1-PE-Cy7 (10F.9G2), and anti-I-Ab-PerCP (M5/114.15.2); all of them obtained from BioLegend (San Diego, CA). A percentage of infection and immunophenotyping were performed by using a FACSCanto II flow cytometer (BD Biosciences, CA), and collected data was analysed using the FlowJo software (Tree Star).

### 2.3. Determination of Transcriptional Levels of Cytokine by Real-Time RT-PCR

To quantify the levels of cytokine transcripts in DCs, RNA was extracted with TRIzol reagent and qRT-PCR was performed for p19 (IL-23), p35 (IL-12), p40 (common subunit for IL23 and IL-12), TGF-*β*, and IL-10 as described before [37]. Briefly, cells were lysed and RNA was extracted using the TRIzol reagent. cDNA (1 mg) was synthesized using oligonucleotides and the M-MLV reverse transcriptase (Promega) according to the manufacturer's instructions. A 20 *μ*l real-time qPCR reaction included 1 *μ*l cDNA, 10 *μ*l Brilliant SYBR Green QRT-PCR Master Mix (Stratagene), and primers and water as indicated by the manufacturer's recommendations. PCR was carried out for 40 cycles with 95°C melting (30 s), 60°C annealing (45 s), and 72°C extension (40 s). All reactions were performed on a Stratagene Mx3000P. Primer sequences were as follows: *p19* forward 5′-TGC TGG ATT GCA GAG CAG TAA-3′ and *p19* reverse 5′-GCA TGC AGA GAT TCC GAG AGA-3′; *p40* forward 5′-ACA GCA CCA GCT TCT TCA TCA G-3′ and *p40* reverse 5′-TCT TCA AAG GCT TCA TCT GCA A-3′; *p35* forward 5′-CAC CCT TGC CCT CCT AAA CC-3′ and *p35* reverse 5′-CAC CTG GCA GGT CCA GAG A-3′; *il10* forward 5′-GAA GAC AAT AAC TGC ACC CA-3′ and *il10* reverse 5′-CAA CCC AAG TAA CCC AAG TC-3′; *tgfb1* forward 5′-TGC GCT TGC AGA GAT TAA AA-3′ and *tgfb1* reverse 5′-CTG CCG TAC AAC TCC AGT GA-3′; and *gapdh* forward 5′-TCC GTG TTC CTA CCC CCA ATG-3′; *gapdh* reverse 5′-GAG TGG GAG TTG CTG TTG AAG-3′. For relative quantification, mRNA expression in each sample was normalized with the *gapdh* mRNA levels using the ddCT method as previously described [39].

### 2.4. Analysis of CD4^+^ T-Cell Differentiation *In Vitro*

T-cell differentiation was analysed as described before [40]. Briefly, naive CD4^+^ CD25^−^ T-cells were obtained at >98% purity by cell sorting on a FACS-ARIA II (BD Biosciences, San Jose, CA) and cocultured with transduced DCs at a 5 : 1 (T : DC) ratio on U-bottom 96-well plates in the presence of 1 *μ*g/ml soluble anti-CD3*ε* monoclonal antibody (mAb) for 5 d. For Th1/Th17 analysis, cells were stimulated with 50 ng/ml PMA and 1 *μ*g/ml ionomycin in the presence of 5 *μ*g/ml brefeldin A during the last 4 h. To assess the intracellular cytokine production, cells were stained with PerCP-conjugated anti-CD4 mAb, fixed with 1% formaldehyde, permeabilized with permeabilizing solution (3% BSA and 0.5% saponin in PBS), and then stained with PE-conjugated anti-IL-17 and allophycocyanin-conjugated anti-IFN-*γ* mAbs. For Treg analysis, cells were stained with PerCP-conjugated anti-CD4, fixed and permeabilized using forkhead box P3 (Foxp3) staining kit (eBioscience, San Diego, CA), and then stained with allophycocyanin-conjugated anti-Foxp3 mAb. Cytokine production and Foxp3 expression were analysed by flow cytometry on a FACS-CANTO II (BD). All cytokines and mAbs were purchased from BD, except for anti-Foxp3 mAb (eBioscience).

### 2.5. Collagen-Induced Arthritis Induction and Evaluation

To induce CIA, 7- to 8-week-old male DBA1/J mice were immunized with type II collagen (CII) as described before [41]. Briefly, bovine CII (Chondrex Inc., Redmond, WA) was dissolved in 0.05 M acetic acid at a concentration of 2 mg/ml by stirring overnight at 4°C and emulsified in an equal volume of complete Freund's adjuvant (for primary immunization) or with incomplete Freund's adjuvant (for secondary immunization). We immunized the mice intradermally at the base of the tail with 100 *μ*g of CII at day 0, and a boost of 100 *μ*g of CII was subcutaneously administered at day 14. To determine the disease severity, macroscopic scale ranging from 0 to 4 was used per paw, where 0 = normal, 1 = detectable arthritis with erythema, 2 = substantial swelling and redness, 3 = severe swelling and redness from joint to digit, and 4 = maximal swelling and deformity with ankylosis. The disease score was expressed as a cumulative value for all paws, with a maximum possible score of 16 per mouse. In some experiments, tolerogenic DCs were i.v. transferred into CIA mice. According to the range of the number of tolerogenic DCs transferred into CIA mice in other studies (5 × 10^5^–5 × 10^6^ DCs per animal) [42–46], we choose the highest number of DCs to evaluate the therapeutic potential of STAT3ca and/or I*κ*B*α*SR transduction (5 × 10^6^ DCs per animal). All procedures performed in animals were approved by and complied with regulations of the Institutional Animal Care and Use Committee at Fundación Ciencia & Vida.

### 2.6. Statistical Analysis

All values were expressed as mean ± SEM. Differences in means between two groups were analysed by 2-tailed Student's *t*-test. Comparisons between more than two experimental groups were performed by one-way ANOVA followed by the multicomparison Tukey's post hoc test. The progression of CIA severity curves and the extent of the therapeutic effect exerted by different DC-based therapies were compared with a nonparametric Mann–Whitney rank-sum two-tailed *U* test. *p* value ≤0.05 was considered significant. Analyses were performed with GraphPad Prism 6 software.

## 3. Results and Discussion

**Figure 1:**
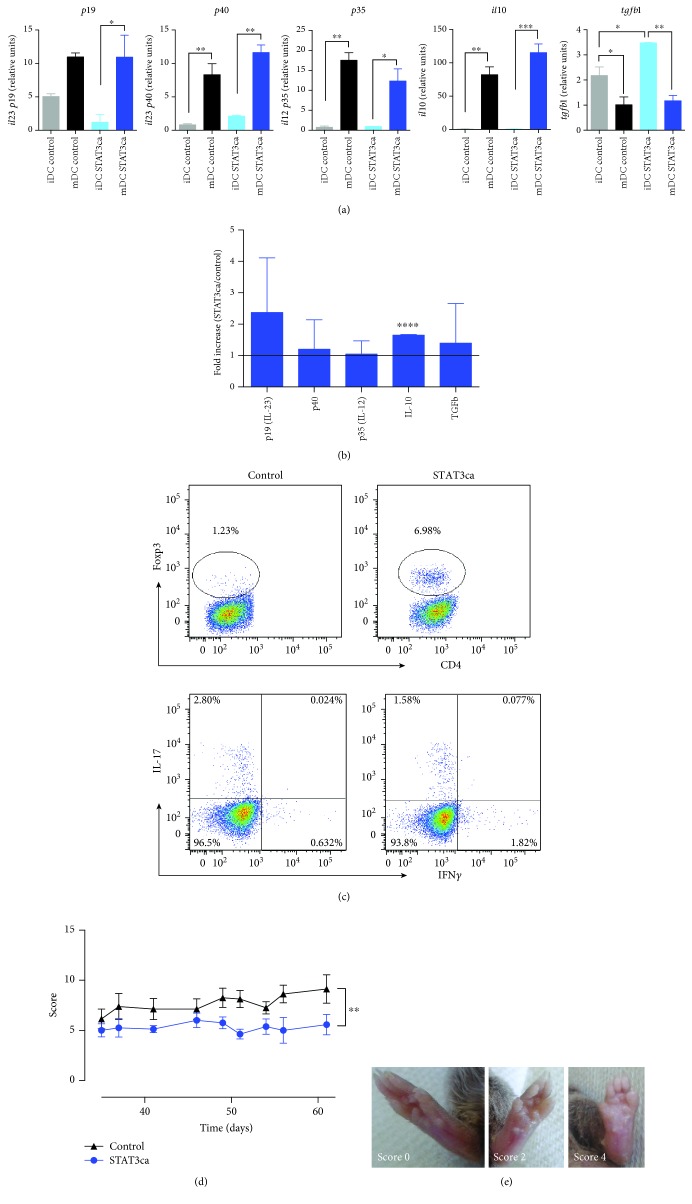
Permanent activation of STAT3 in DCs induces a tolerogenic profile with therapeutic effects in CIA. Bone marrow-derived precursors from DBA/1J mice were cultured in conditions to differentiate to DCs and transduced on days 4 and 5 with lentiviral vectors (MOI 10) encoding for STAT3ca or with empty pLVX vectors (control) in the presence of polybrene. At day 6, DCs were either left without treatment (iDC) or treated with 500 ng/ml LPS for 24 h (mDC). (a, b) Levels of mRNA encoding for cytokines were evaluated in transduced DCs by quantitative real-time RT-PCR and normalized with the levels of *gapdh* mRNA. (a) Levels of mRNA encoding for different cytokines are represented as relative units. Values are mean ± SD from three or more independent experiments carried out in duplicate. ^∗^*p* < 0.05, ^∗∗^*p* < 0.01, and ^∗∗∗^*p* < 0.001 by one-way ANOVA followed by Tukey's post hoc test. (b) Data presented as the fold increase of mRNA levels in mDCs relative to mDCs transduced with control vectors. Values are mean ± SD from three or more independent experiments carried out in duplicate. Ratio = 1, which indicates no differences between cytokine mRNA levels in mDCs transduced with STAT3ca and in mDCs transduced with control vectors, is represented by a dotted line. ^∗∗∗∗^*p* < 0.0001 by unpaired two-tailed Student's *t*-test. (c) After lentiviral transduction, DCs were cocultured with naive CD4^+^ T-cells (at DC : T-cell ratio of 1 : 5) in the presence of anti-CD3*ε* antibody and incubated for 5 d. Afterward, the percentage of Treg cells was evaluated by intracellular immunostaining of Foxp3 in the CD4^+^ T-cell population. To determine the percentage of Th1 and Th17 lymphocytes, T lymphocytes were restimulated with PMA and ionomycin in the presence of brefeldin A for the last 4 h of culture and the extent of IFN-*γ* and IL-17 was assessed by intracellular cytokine immunostaining and analysed by flow cytometry in the CD4^+^-gated population. Dot plots of Foxp3 versus CD4 (top panels) and IFN-*γ* versus IL-17 in the CD4^+^ population (bottom panels) are shown. Representative data from three independent experiments is shown. The percentage of cells present in each quadrant is indicated. (d) After transduction, mDCs were loaded with 40 *μ*g/ml of CII for 18 h and then intravenously administered (5 × 10^6^ transduced DCs/mouse) to DBA/1J mice at day 35 after CIA induction. The extent of disease severity was evaluated along with the disease development as a score described in Materials and Methods. Data represents mean ± SD from eight animals per group. ^∗∗^*p* < 0.01 by Mann–Whitney *U* test. (e) Representative photos of mouse paws displaying no inflammation (score 0, left image), mild inflammation (score 2, middle image), and strong inflammation (score 4, right image).

**Figure 2:**
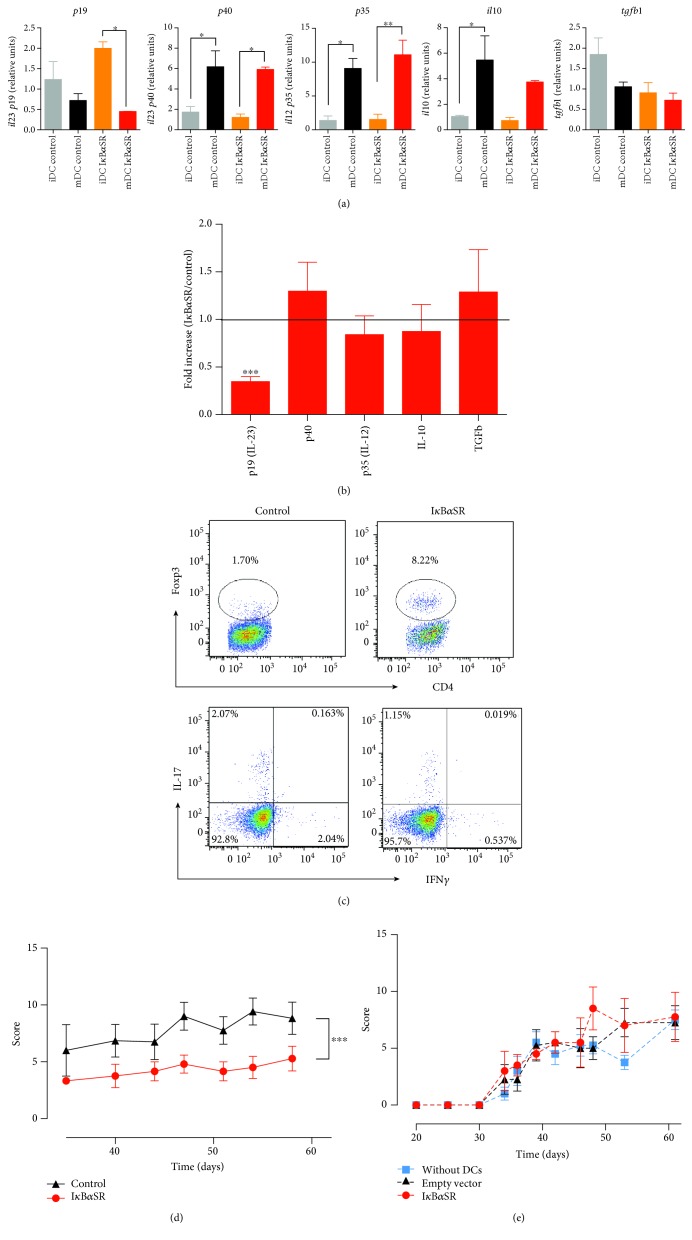
Constitutive repression of NF-*κ*B favours the acquisition of tolerogenic properties by DCs, which attenuate CIA development. Bone marrow-derived precursors from DBA/1J mice were cultured in conditions to differentiate to DCs and transduced on days 4 and 5 with lentiviral vectors (MOI 10) encoding for I*κ*B*α*SR or with empty PGK-Cre vectors (control) in the presence of polybrene. At day 6, DCs were either left without treatment (iDC) or treated with 500 ng/ml LPS for 24 h (mDC). (a, b) Levels of mRNA encoding for cytokines were evaluated in transduced DCs by quantitative real-time RT-PCR and normalized with the levels of *gapdh* mRNA. (a) Levels of mRNA encoding for different cytokines are represented as relative units. Values are mean ± SD from three or more independent experiments carried out in duplicate. ^∗^*p* < 0.05, ^∗∗^*p* < 0.01, and ^∗∗∗^*p* < 0.001 by one-way ANOVA followed by Tukey's post hoc test. (b) Data presented as the fold increase of mRNA levels in mDCs relative to mDCs transduced with control vectors. Values are mean ± SD from three or more independent experiments carried out in duplicate. Ratio = 1, which indicates no differences between cytokine mRNA levels in mDCs transduced with I*κ*B*α*SR and in mDCs transduced with control vectors, is represented by a dotted line. ^∗∗∗^*p* < 0.001 by unpaired two-tailed Student's *t*-test. (c) After lentiviral transduction, DCs were cocultured with naive CD4^+^ T lymphocytes (at DC : T-cell ratio of 1 : 5) in the presence of anti-CD3*ε* antibody and incubated for 5 d. Afterward, the percentage of Treg lymphocytes was evaluated by intracellular immunostaining of Foxp3 in the CD4^+^ gate. To determine the percentage of Th1 and Th17 lymphocytes, cells were restimulated with PMA and ionomycin in the presence of brefeldin A for the last 4 h of culture and the extent of IFN-*γ* and IL-17 was assessed by intracellular cytokine immunostaining and quantified by flow cytometry in the CD4^+^-gated population. Dot plots of Foxp3 versus CD4 (top panels) and IFN-*γ* versus IL-17 in the CD4^+^ population (bottom panels) are shown. Representative data from three independent experiments is shown. The percentage of cells present in each quadrant is indicated. (d) After transduction, mDCs were loaded with 40 *μ*g/ml of CII for 18 h and then intravenously transferred (5 × 10^6^ transduced DCs/mouse) into DBA/1J mice at day 35 after CIA induction. The disease score was evaluated along with CIA development as described in Materials and Methods. Data represents mean ± SD from eight animals per group. ^∗∗∗^*p* < 0.001 by Mann–Whitney *U* test. (e) After transduction, DCs were not pulsed with any exogenous antigen and intravenously administered (5 × 10^6^ transduced DCs per animal) to DBA/1J mice at day 35 after CIA induction. Experimental groups receiving DCs transduced with I*κ*B*α*SR or with the empty PGK-Cre control vector (empty vector) were also compared with a group of mice that did not receive DCs (without DCs). The disease score was evaluated along with CIA development as described in Materials and Methods. Data represents mean ± SD from four animals per group. No significant differences were detected between different experimental groups.

To determine the potential of targeting STAT3 signalling or NF-*κ*B activation to generate permanent tolerogenic DCs, we first transduced bone marrow-derived immature DCs (iDCs) with lentiviral vectors encoding a constitutively active version of STAT3 (STAT3ca) or a nonphosphorylatable version of the NF-*κ*B inhibitor I*κ*B*α* (I*κ*B*α* superrepressor (I*κ*B*α*SR)), and then, they were either left without treatment or treated with LPS for 24 h to induce maturation (mDCs) and the production of proinflammatory and anti-inflammatory cytokines was assessed. LPS-induced maturation promoted an increased transcription of *il10*, *il12*, and *il23*, whilst decreasing the levels of *tgfb1* mRNA when transduced with either STAT3ca or empty control vectors (Figure 1(a)). Similarly, when transduced with I*κ*B*α*SR or the associated empty control vector, transcriptional levels of both *il12* subunits *p40* and *p35* were significantly increased upon LPS treatment; however, *tgfb1* mRNA levels did not change (Figure 2(a)). Furthermore, whereas transcriptional levels of *il23* (*p19*) did not change upon maturation in DCs transduced with control vectors, they were significantly reduced in I*κ*B*α*SR-transduced DCs after LPS treatment (Figure 2(a)). In addition, transcriptional levels of *il10* were enhanced upon LPS-induced maturation only when DCs were transduced with the empty control vector, but not when transduced with I*κ*B*α*SR (Figure 2(a)). Thus, I*κ*B*α*SR-transduced DCs present a significant reduction of *il23 p19* transcripts exclusively after LPS treatment, whereas STAT3ca-transduced DCs display an interesting trend increasing *il10* mRNA levels after LPS-induced maturation (Figures 1(a) and 2(a)). To gain a deeper insight of the effect of STAT3ca transduction and I*κ*B*α*SR transduction in the extent of proinflammatory and anti-inflammatory cytokine transcription in DCs upon LPS treatment, we next analysed the ratio of cytokine transcripts observed in mDCs transduced with STAT3ca or I*κ*B*α*SR relative to those mRNA levels detected when transduced with control vectors. The results show that constitutive activation of STAT3 induced a significant increase in *il10* levels (Figure 1(b)), whilst the permanent repression of NF-*κ*B activation resulted in a significant reduction of *il23* levels (Figure 2(b)). To assess the tolerogenic activity of STAT3ca-transduced and I*κ*B*α*SR-transduced DCs *in vitro*, we next performed coculture experiments in which naive CD4^+^ T-cells were cocultured with transduced DCs in the presence of anti-CD3*ε* mAb as a polyclonal T lymphocyte activation stimulus and the extent of Treg, Th1, and Th17 differentiation was assessed after 5 d. Importantly, the results show that both STAT3ca transduction and I*κ*B*α*SR transduction of DCs induced a marked increase in the generation of Treg lymphocytes and without effects in the extent of Th1 and Th17 differentiation in comparison with DCs transduced with control vectors (Figures 1(c) and 2(c)), indicating that constitutive activation of STAT3 and permanent NF-*κ*B repression contribute to the tolerogenic potential of DCs. Together, these results suggest that both STAT3 activation and NF-*κ*B inhibition contribute to induce a tolerogenic behaviour in DCs in different ways. To assess the therapeutic effect of these DCs with tolerogenic profiles, we next used an animal model equivalent to rheumatoid arthritis, an autoimmune pathology involving a relevant role of DCs in the induction of Th1- and Th17-mediated inflammation [47]. Accordingly, we use the mouse model of CIA, which involves an autoimmune response to collagen driven by autoreactive Th1 and Th17 lymphocytes [26]. To this end, STAT3ca-transduced DCs or I*κ*B*α*SR-transduced DCs were loaded with type II collagen (CII) as an autoantigen, and then intravenously administered in mice undergoing CIA, and the disease severity was determined by scoring the extent of inflammation of mouse paws (Figure 1(e)) throughout the time course of the disease development. Importantly, the results show that the constitutive STAT3 activation and the permanent NF-*κ*B repression in DCs exerted a significant therapeutic effect reducing CIA manifestation (Figures 1(d) and 2(d)). To compare the therapeutic potential of tolerogenic DCs generated by lentiviral transduction with that of tolerogenic DCs generated by treatment with immunosuppressive drugs, we performed control experiments in which CIA mice received the intravenous transfer of CII-pulsed tolerogenic DCs (5 × 10^6^ cells/animal) generated by STAT3ca transduction or by incubation with vitamin D3 [48, 49]. The results show that the transfer of vitamin D3-treated DCs had a significant effect attenuating disease severity. However, CIA mice receiving the transfer of STAT3ca-transduced DCs displayed a significantly stronger reduction of CIA manifestation in comparison with those mice receiving DCs treated with vitamin D3 (data not shown). Although a number of studies have shown that tolerogenic DCs need to be pulsed with a disease-relevant autoantigen to induce an anti-inflammatory effect in autoimmune disorders, another group of studies has suggested that nonloaded tolerogenic DCs might take up relevant autoantigens *in vivo* and exert a therapeutic effect dampening inflammation [50, 51]. Thereby, whether the loading of tolerogenic DCs with autoantigens is necessary or not to attenuate inflammation in autoimmune disorders remains controversial. For this reason, we next attempted to assess the anti-inflammatory potential of nonantigen-pulsed tolerogenic DCs. For this purpose, we compared the severity of CIA manifestation in mice that did not receive exogenous DCs and in mice that received nonantigen-pulsed I*κ*B*α*SR-transduced DCs or that received the transfer of nonantigen-pulsed DCs transduced with control vectors. The results show no significant differences in CIA manifestation among these experimental groups (Figure 2(e)), thus indicating that the loading of tolerogenic DCs with a disease-relevant autoantigen (CII) is necessary to induce a therapeutic effect under the experimental conditions used in this study.

**Figure 3:**
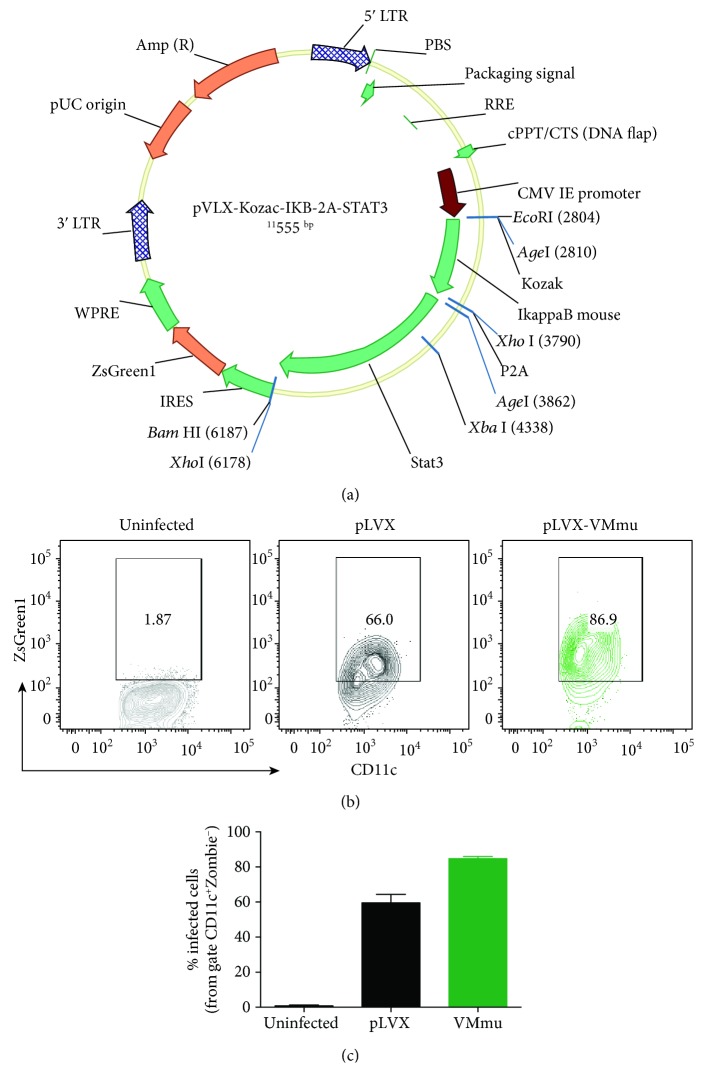
Generation of a master viral vector encoding murine STAT3ca and I*κ*B*α*SR (pLVX-VMmu). (a) Lentiviral genome maps for pLVX-VMmu vector containing the pLVX backbone encoding I*κ*B*α*SR and STAT3ca genes separated by a P2A sequence. As a reporter gene, the green fluorescent protein (ZsGreen1) was inserted at 3′ after an IRES sequence. These three genes were inserted under the control of the CMV promoter. Kozak sequence was also included where indicated. (b, c) Bone marrow-derived precursors from DBA/1J mice were cultured in conditions to differentiate to DCs in the absence of lentiviral treatment (grey) or transduced at days 4 and 5 with viral vectors (MOI 10) encoding for pLVX-VMmu (green) or with the empty control vector pLVX (black) in the presence of polybrene. After 24 h, the efficiency of lentiviral transduction was determined as the extent of ZsGreen1 expression by flow cytometry in the CD11c^+^ population. (b) Representative contour plots of CD11c versus ZsGreen1 expression. The percentage of CD11c^+^ cells expressing ZsGreen1 is indicated in the corresponding region. (c) Quantification of the efficiency of DC transduction determined as the percentage of ZsGreen1^+^ cells from the CD11c^+^-gated population. The analysis was carried out excluding dead cells by selecting only cells with negative staining for Zombie Aqua. Values are mean ± SD from three independent experiments carried out in triplicate. No differences were detected between the efficiency of DC transduction with pLVX and with pLVX-VMmu.

**Figure 4:**
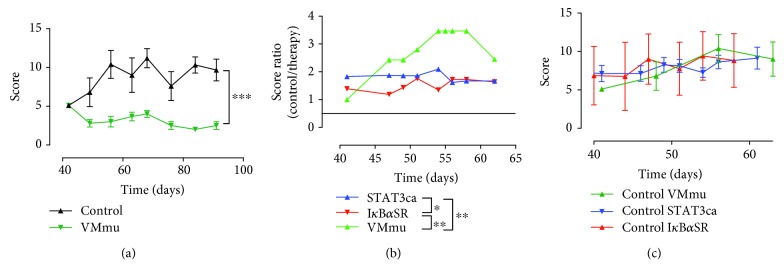
Simultaneous transduction of DCs with STAT3ca and I*κ*B*α*SR exerts a more pronounced attenuation of CIA manifestation. (a) mDCs from DBA/1J mice were transduced with pLVX-VMmu (green) or with the control empty vector pLVX (black) as indicated in Figure 3, pulsed with 40 *μ*g/ml CII for 18 h, and then intravenously transferred (5 × 10^6^ transduced DCs/animal) into DBA/1J mice at day 40 after CIA induction. The disease score was evaluated along with CIA development as described in Materials and Methods. Data represents mean ± SD from at least eight animals per experimental group. ^∗∗∗^*p* < 0.001 by Mann–Whitney *U* test. (b) Comparison of the therapeutic effect induced by the intravenous transfer of DCs transduced with different lentiviral vectors shown in Figures 1(d), 2(d), and 4(a) throughout the time course of CIA development. Values represent the ratios of average disease score obtained with the control and average disease score obtained with the therapy. Dotted line represents ratio = 1, which indicates no therapeutic effect. ^∗^*p* < 0.05 and ^∗∗^*p* < 0.01 by Mann–Whitney *U* test. (c) Disease severity of control groups from Figures 1(d), 2(d), and 4(a) was compared along with CIA development. Values are mean ± SD from at least eight animals per experimental group. No significant differences were found by Mann–Whitney *U* test.

Since we observed different cytokine profiles in DCs transduced with STAT3ca or with I*κ*B*α*SR and both treatments induced significant therapeutic effects attenuating CIA development, we reasoned that stimulation of STAT3 signalling and inhibition of NF-*κ*B activation should be complementary in promoting a tolerogenic profile in DCs. Supporting this notion, previous studies have suggested that STAT3 and canonical NF-*κ*B are involved in different signalling pathways regulating the tolerogenic phenotype of DCs [52]. According to this idea, we next generated viral vectors encoding both STAT3ca and I*κ*B*α*SR together (Figure 3(a)). Of note, using this master viral vector encoding murine STAT3ca and I*κ*B*α*SR (pLVX-VMmu), we obtained 60-90% transduction efficiency in DCs (Figures 3(b) and 3(c)). Afterward, the therapeutic potential of tolerogenic DCs generated by the simultaneous transduction of STAT3ca and I*κ*B*α*SR was assessed in CIA. The results show that simultaneous transduction with STAT3ca and I*κ*B*α*SR exerted a more pronounced attenuation of CIA manifestation in comparison with the effect exerted by single transduction of DCs with STAT3ca or I*κ*B*α*SR separately (Figures 4(a) and 4(b)). Since the therapeutic potential of VMmu-, STAT3ca-, and I*κ*B*α*SR-transduced DCs was determined in separated independent experiments, we sought to evaluate whether the extent of CIA severity was equivalent in these different rounds of experiments. Accordingly, we compared CIA development of control groups from these different experiments. This analysis shows no differences between the developments of CIA in the different rounds of experiments (Figure 4(c)), thereby ruling out the possibility that the stronger therapeutic effect observed for VMmu-transduced DCs was just due to a different extent of CIA development in the control group.

A major issue in Treg-based suppressive immunotherapy in RA has been shown to be the instability of the therapeutic Treg lymphocytes when exposed to an inflammatory environment *in vivo*. In this regard, it has been shown that ex vivo generated Treg lymphocytes transdifferentiate into pathogenic Th17 lymphocytes when transferred in mice undergoing CIA, an effect exerted by IL-6 derived from synovial fibroblasts [53]. Interestingly, it has been shown that IL-6-mediated transdifferentiation of Treg lymphocytes is mediated by STAT3 activation, which inhibits Foxp3 expression and reciprocally favours IL-17 expression. Of note, a recent study has shown that IL-6-mediated lymphocyte transdifferentiation might be attenuated by the activity of the phosphatase PTPN2, which dephosphorylates and inactivates STAT3 in Treg lymphocytes [54]. The problem of Treg instability has been overcome by viral transduction of the master transcription factor Foxp3, which controls the Treg immunosuppressive phenotype. In this regard, the generation of antigen-specific Treg lymphocytes with chimeric antigen receptors recognizing myelin oligodendrocyte glycoprotein and the carcinoembryonic antigen are two examples of immunosuppressive therapies based on the generation of Treg lymphocytes by ex vivo viral transduction which have shown successful therapeutic effects in mouse models of multiple sclerosis and inflammatory bowel diseases, respectively [55, 56].

Similar to Treg-based immunotherapy, a number of studies have shown successful therapeutic effects in animal models of autoimmune disorders and inflammatory conditions when treated with tolerogenic DCs generated by lentiviral manipulation of gene expression. For instance, the *ex vivo* transduction of DCs with the vasoactive intestinal peptide, which displays anti-inflammatory activity, has shown to generate stable tolerogenic DCs that induce significant therapeutic effects reducing inflammation in animal models of multiple sclerosis and sepsis [30]. In another study, authors generated tolerogenic DCs *ex vivo* by lentiviral transduction of interference RNA for *cd40* and *il23*, which induce potent therapeutic effects dampening disease manifestation in a mouse model of multiple sclerosis [31]. Similarly, in a recent study, the authors show that knocking down CD40 expression by lentiviral transduction generates potent tolerogenic DCs which exerted significant improvement of disease manifestation in a mouse model of autoimmune diabetes [32]. Importantly, lentiviral generation of tolerogenic DCs has shown promising therapeutic effects not only in animal models of autoimmune disorders but also in allograft rejection. In this regard, a recent study shows how the knockdown of RelA, a positive regulator of NF-*κ*B activation, attenuates the corneal allograft rejection in an animal model of allogeneic corneal transplant [33]. In the present work, we show that, by mean of lentiviral transduction, the permanent STAT3 activity or the constitutive NF-*κ*B repression induces the generation of tolerogenic DCs with therapeutic activity in a mouse model of RA. Furthermore, our results show that the simultaneous delivery of both features together in DCs, the permanent STAT3 activity and the constitutive NF-*κ*B inhibition, results in the generation of DCs with stronger therapeutic potential when compared with DCs generated only with constitutive NF-*κ*B inhibition or permanent STAT3 activity. These findings show STAT3 and NF-*κ*B as two important and complementary regulators of the tolerogenic behaviour of DCs, which should be considered as molecular targets in the design of DC-based suppressive immunotherapies for the treatment of autoimmune disorders.

## 4. Conclusions

Our results show that the constitutive activity of STAT3 in DCs results in increased IL-10 production and the acquisition of tolerogenic features with the ability to reduce significantly the development of CIA. On the other hand, the results show that the permanent inhibition of NF-*κ*B in DCs induced a significant attenuation in the expression of IL-23, promoting a tolerogenic behaviour that dampens inflammation when transferred into mice undergoing CIA. Since the results showed a selective effect of STAT3 activity favouring IL-10 and of NF-*κ*B inhibition in attenuating IL-23, we reasoned that constitutive STAT3 activity would increase Treg generation, whilst permanent NF-*κ*B inhibition would reduce Th17 differentiation. Accordingly, we found that constitutive STAT3 activity and permanent NF-*κ*B inhibition exerted complementary effects inducing tolerogenic features in DCs, thus increasing their therapeutic potential when STAT3ca and I*κ*B*α*SR were delivered together in DCs in comparison with when transduced each alone in DCs. These findings show STAT3 and NF-*κ*B as two complementary molecular targets to be considered in the design of DC-based tolerogenic immunotherapies for the treatment of RA and other autoimmune disorders.

## Data Availability

All relevant data is within the paper. For access to raw data, readers can require it to the corresponding author.
